# The utility of neutrophil gelatinase-associated Lipocalin (NGAL) as a marker of acute kidney injury (AKI) in critically ill patients

**DOI:** 10.1186/s40364-019-0155-1

**Published:** 2019-02-22

**Authors:** Shabnum Khawaja, Lena Jafri, Imran Siddiqui, Madiha Hashmi, Farooq Ghani

**Affiliations:** 1 Department of Pathology& Laboratory Medicine, Aga Khan University Hospital (AKUH), Stadium Road, P.O. Box 3500, Karachi, 74800 Pakistan; 2 Department of Anesthesiology, Aga Khan University Hospital(AKUH)Stadium Road, P.O. Box 3500, Karachi, 74800 Pakistan

**Keywords:** Acute kidney injury (AKI), Sepsis, Plasma neutrophil gelatinase associated Lipocalin (pNGAL), Intensive care unit (ICU)

## Abstract

In current clinical practice, Serum Creatinine (SCr) is a commonly used marker for the diagnosis of acute kidney injury (AKI). Unfortunately, due to a delayed increase in SCr, it is unable to accurately estimate the timing of the injury. The purpose of this study was to assess the ability of plasma neutrophil gelatinase-associated lipocalin (pNGAL) to predict AKI in critically ill adult patients**.** The study was conducted at the Section of Chemical Pathology, Department of Pathology& Laboratory Medicine in collaboration with Department of Anesthesiology, at Aga Khan University Hospital in Karachi, Pakistan. Subjects in the age groups of18 to 60, that were admitted into the intensive care unit (ICU) with suspected sepsis were enrolled in this study.AKI was labeled by using Risk-Injury-Failure-loss-End Stage (RIFLE) criteria. Forty-eight patients, mean age being 46.5 ± 16.3, were recruited over a nine-month period. Multiple blood samples were collected from each patient at 12 h, 24 h, and 48 h. A total of 52.1% (*n* = 24) of ICU patients suspected of sepsis had developed AKI. Baseline characteristics of subjects with AKI were compared to those without AKI. Statistically significant difference was noted in gender (*p*-value< 0.05) and pNGAL (*p*-value< 0.001). However, no significant differences were seen with respect to age, in patients with and without AKI. The area under the curve (AUC) at12hr was 0.82 (95% CI 0.68–0.96) with a sensitivity of 70.8% and specificity of 90.9%.While AUCs at 24 h was 0.86(95% CI 0.74–0.97) with a sensitivity of 78.5% and specificity of 88.8%. Furthermore, there was a positive correlation between pNGAL and the length of ICU stay (*r* = 0.98). Non-survivors or expired patients had higher median pNGAL170 (202–117) ng/ml as compared to survivors 123(170–91) ng/ml**.** In conclusion, pNGAL is an early predictor of AKI in a heterogeneous adult ICU population. Plasma NGAL allows the diagnosis of AKI 48 h prior to a clinical diagnosis based on RIFLE criteria. Early identification of high-risk AKI in patients may allow earlier initiation of therapies and improve patient outcome.

## Introduction

Acute kidney injury (AKI) is a rapid loss of kidney function following failure to maintain fluid, acid-balance, and electrolyte homeostasis [1]. Acute kidney injury alone complicates7.2 to 20% of hospitalized patients and 13–78% of intensive care unit (ICU) patients [2–8]. Critically ill patients with concomitant AKI have a poor prognosis and a high mortality rate. Sepsis is a common cause of AKI in critically ill patients and various observational studies have found that sepsis contributes 30–50% of all AKI cases [9].

In the last few decades, serum Creatinine (SCr) is used for the diagnosis of AKI in common practice, but SCr is a functional marker and not an ideal biomarker to identify AKI. The values of SCr are influenced by multiple non-renal factors, like age, gender, muscle mass and metabolism, dietary habits, medications, and hydration status [9]. There is a considerable delay in increase of SCr after AKI and it does not reflect the actual decrease in glomerular filtration rate (GFR). Additionally, SCr may not rise until more than half of the kidney function has been lost. Delay in prompt diagnosis and management of AKI may cause irreversible kidney damage. RIFLE criterion was proposed by Acute Dialysis Outcome Initiative(ADQI) group in order to have a uniform standard for diagnosis and classification of AKI, including Risk(R), Injury (I), Failure (F), Loss (L), and End-Stage (E) renal disease as a tool for qualifying and quantifying the severity of AKI [10]. The RIFLE based on either two-fold increase in the SCr from baseline, or GFR decrease by 50%, or urine output < 0.5 ml/kg per hour for 12 h. Many studies in the literature have applied SCr criteria (RIFLE-SCr), but there are some inherent limitations. First, it is based on relative changes in serum or plasma Cr, due to varying extrarenal clearance; Cr is a late and unreliable responder to GFR alterations. Second, Cr is a small molecule (113 Da) and may pass through the glomerular filter freely even when glomerular pores are moderately narrowed [11]

There is a need for a reliable AKI biomarker, which should be sensitive, specific, depict early change following kidney injury, easy to measure, and does not require administration of an exogenous substance. Many new promising AKI biomarkers have been identified and one such promising biomarker is plasma Neutrophil Gelatinase-Associated Lipocalin (pNGAL) [12]. Numerous studies have been done on this biomarker, but it is still controversial to use this biomarker in the clinical setting as an AKI marker. Metanalysis done by An Zhang and his colleagues (2016) stated that” Utility of NGAL to predict the occurrence of AKI in septic patients remains controversial” [13]. Therefore, the objective of this study is to assess the ability of pNGAL to predict early AKI in critically ill adult patients presenting with sepsis.

## Material and methods

### Patient recruitment

A cross-sectional study was conducted at the Section of Chemical Pathology, Department of Pathology & Laboratory Medicine in collaboration with the Department of Anesthesiology at Aga Khan University Hospital in Karachi, Pakistan during December 2014 to August 2015. Patient clinical details along with demographics and relevant biochemical data were recorded on a structured proforma within 12 h of admission to intensive care unit (ICU).

### Inclusion criterion

All subjects that are between the ages of 18–60, admitted to the ICU at Aga Khan University Hospital with suspected sepsis were included in the study. Sepsis was defined as per systemic inflammatory response syndrome (SIRS) criterion if patients presented with: body temperature > 38 °C (100.4 °F) or < 36 °C (96.8 °F),heart rate > 90 beats per minute, respiratory rate > 20 breaths per minute or arterial carbon dioxide tension (PaCO _2_) < 32 mmHg, Abnormal white blood cell count (> 12,000/μL or < 4000/μL or > 10% immature and white blood cell count > 12,000 × 10 [8]/L or < 4000 × 10 [8]/L. [11, 12]

### Prediction of AKI and calculation of baseline serum creatinine

Using Risk-Injury-Failure-Loss-End Stage (RIFLE-SCr) proposed by the Acute Dialysis Quality Initiative (ADQI) group [14]. A twofold increase in SCr from baseline value was considered as AKI [15]. Cut off 150 ng/ml of Plasma NGAL used for the Prediction of Acute kidney injury [13, 16].

Patient’s baseline serum Creatinine (bSCr) levels were calculated by using the Modification of Diet in Renal Disease (MDRD) equation proposed by the Acute Dialysis Quality Initiative (ADQI) working group by using formula [17]:

Serum Creatinine = (75/186× (age^− 0.203^) × (0.742 if female) × (1.21 if black)]) ^− 0.887^

### Exclusion criterion

Pregnant females, patients with known chronic kidney disease, patients on hemodialysis, renal transplant recipients, and those with known kidney malignancy were excluded**.** Death of patients or early ICU discharged patients was also excluded from this study.

### Blood sampling and biochemical analysis

Serial blood sampling was done for pNGAL in the following manner: first within 12 h of admission to ICU, second after 24 h, and a third sample was drawn after 48 h of the first sample. Three to four milliliters of blood was drawn each time in ethylenediaminetetraacetic-acid (EDTA) vacuutainer. Blood samples were centrifuged at 3000 rpm for 15 min, aliquoted (for what> be specific), and stored at − 80 °C until analysis. The pNGAL was analyzed by fluorescence immunoassay on Triage® Meter Proby Alere Diagnostics point of care analyzer, CA, US. Two levels of manufacturer-provided quality controls (low and high) were run with each batch of analysis.

### Statistical analysis

Data was analyzed using the Statistical Package for the Social Sciences (SPSS version 19.0). Frequency and percentages were calculated for gender. Mean, standard deviation, and median with interquartile ranges was computed for a continuous variable, the age of the patients, duration of hospital stay, and pNGAL. The pNGAL levels between patients with and without AKI were compared by independent t-test. The diagnostic accuracy of pNGAL to predict AKI in critically ill patients of ICU was assessed by applying Receiver Operator Curve (ROC) analysis and calculating the area under the curve (AUC) at 24 and 48 h. A *p*-value of < 0.05 was considered as statistically significant.

## Results

**Fig. 1:**
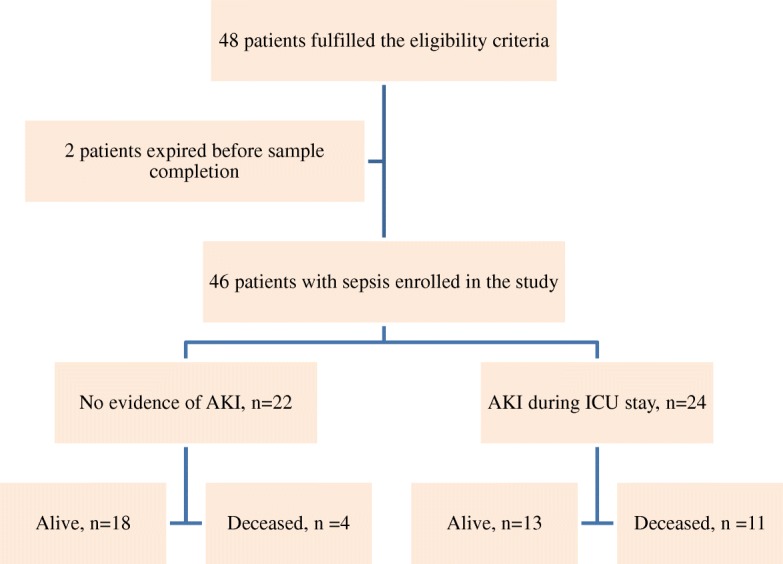
Profile of recruitment and outcome of study group

In this prospective study, adult patients admitted into the ICU with suspicion of sepsis were recruited over a period of nine months. However, two of the 48 patients expired before blood sampling was completed. Forty-six patients fulfilled the criteria for sepsis and were subjected to blood sampling and biomarker analysis (Fig. 1). The mean age of patients (*n* = 46) was 46.5 ± 16.3 years with the majority being males. Mean BMI of the patients was 26.2 ± 4.8 kg/m [9]. Baseline mean SCr of males and females was 1.1 ± 0.1 mg/dl and 0.8 ± 0.7 mg/dl respectively. Table 1 describes the general characteristics and clinical details of the study group. Median ICU stay of the study group was 6 days (4–13 days).

**Table 1:** Demographics and clinical details of patients (*n* = 46) admitted to ICU with sepsis

Characteristics	n (%)
Males n (%)	32 (69%)
**Stratification of subjects as per WHO classification**	
Normal	16 34.8%)
Overweight	23 (50%)
Obese	7 (15.2%)
**Comorbids**	
No Comorbids	20 (43.5%)
DM, HTN and Cardiovascular Disease	16 (32.6%)
Cardiovascular Disease	3 (6.5%)
DM	2 (4.3%)
Pulmonary Disease	2 (4.3%)
Liver Disease	2 (4.3%)
Malignancy	1 (2.2)

Abbreviations: intensive care unit, *ICU* basal metabolic index, *BMI* diabetes mellitus, *DM* hypertension, HTN

**Table 2:** Biochemical characteristics of patients with and without AKI

Variables	Patients with suspected sepsis (*n* = 46)		*p*-value
	AKI *n* = 24	Non-AKI *n* = 22	
Male n (%)	18 (39.1%)	14 (30%)	< 0.05
Mean WBC count × 10 [8]	16.0 ± 7.1	16.7 ± 6.3	> 0.05
Median pNGAL at 12 h (ng/ml)	185 (262–142)	110 (120–70)	< 0.05
Median pNGAL at 24 h (ng/ml)	400 (585–185)	130 (145–80)	< 0.05
Median pNGAL at 48 h (ng/ml)	597 (452–1050)	133 (140–60)	< 0.05
Non-Survivors	11 (45.8%)	4 (16.6%)	> 0.05

Quantitative variables are expressed in mean ± SD or median (IQR) and frequencies as n (%). Abbreviations: acute kidney injury, *AKI* Plasma neutrophil Gelatinase associated Lipocalin, pNGAL, SerumCreatinine, Scr

Plasma NGAL levels increased by 41.3%(*n* = 19) at 12 h, 50%(*n* = 23) at 24 h and 54.3%(*n* = 25) at 48 h of ICU admission. Using RIFLE criteria, a total of 52.1% (*n* = 24) of ICU patients developed AKI during their ICU stay. Out of these pNGAL levels increased by 70.8%(*n* = 17) at 12 h of ICU admission and 79%(n = 19) patients at 24 h of ICU admission. However, 5 patients were mislabeled by pNGAL as AKI. SCr reached endpoint at 48 h of ICU stay with median SCr was 2.3(3.1–2.1) mg/dl at 48 h of ICU admission.

**Fig. 2:**
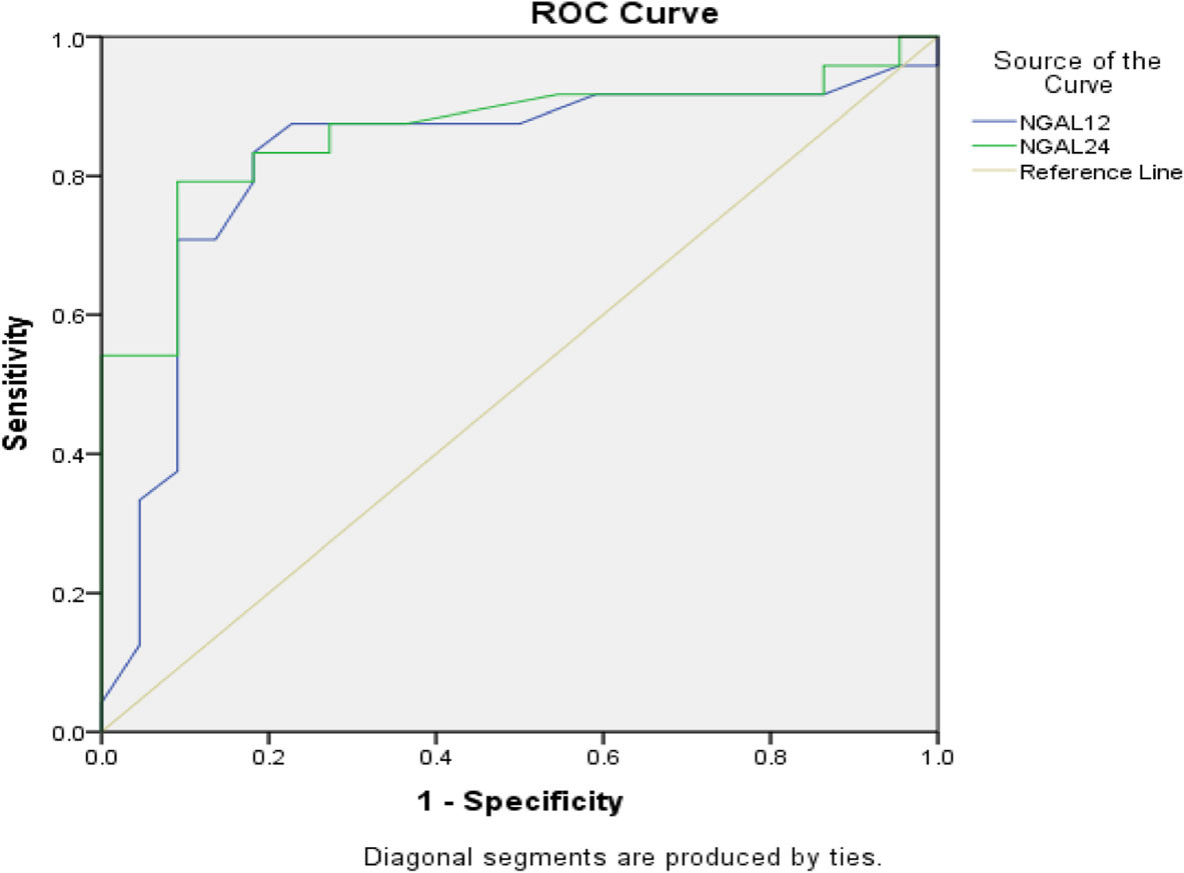
ROC curve of pNGAL at the 12 and 24 h time points

Baseline characteristics of subjects with AKI were compared with those without AKI. Statistically, a significant difference was noted in gender (pvalue< 0.05) and pNGAL (value< 0.001) between patients with (Fig. 2) and without AKI (Table 2). However, no significant differences were seen in age, between patients with and without AKI. AUCs at12 hours was 0.82 (95%CI 0.68–0.96) with sensitivity of 70.8% and specificity of 90.9%. AUCs at 24 h was 0.86(95%CI 0.74–0.97) with the sensitivity of 78.5% and specificity of 88.8%. There was a positive correlation between pNGAL and the duration of the ICU stay (r = 0.98). Mortality rate was 32.6% (*n* = 15), while 67.3%(*n* = 31) of patients were shifted to the wards from the ICU. Amongst expired patients12hour median pNGAL was 170(202–117) ng/ml as compared to survivors which were 123(170–91) ng/ml.

## Discussion

The utilization of pNGAL for the prediction of AKI was studied. All patients that were admitted with suspected sepsis not having AKI on admission were used as the study subjects to analyze p NGAL for the prediction of AKI. It was observed that pNGAL levels were significantly higher in patients who developed AKI in comparison who did not develop AKI (*p*-value< 0.05).This allowed to distinguish AKI patients from non-AKI patients. A similar study performed by Soto and his colleagues (2013), stated that highest median levels of pNGAL seen in patients with AKI (146–174 ng/ml at various points) increased level with AKI severity (207–244 ng/ml) [18]. Another study performed by Usman et al. in 2013 also reported nearly similar median (IQR) of pNGAL of 170 ng/ml (126–274 ng/ml) in patients who develop AKI [19]

There is no consensus about the cutoff value of pNGAL at which AKI diagnosis can be made accurately [20].We used a cutoff of 150 ng/ ml to predict AKI, which was similar cutoff used by Camou et al. in 2013 [16] and Wang et al. [21] in 2014 to predict AKI. A systematic review and meta-analysis done by Zhang et al. [13] which included the studies of Yamashita et al. [22] and Dai et al. [23] reported diagnostic ability of pNGAL with AUCs of 0.94 (95% CI, 0.88–0.97) and 0.92 (95% CI, 0.84–0.96) respectively for plasma NGAL to predict AKI in septic patients similar to our findings. Azrina ralib et al. reported similar AUC for pNGAL for the diagnosis of AKI, 0.81 (95%CI 0.74 to 0.87) [24].

NGAL was originally isolated from secondary granules of human neutrophils as a 25 KDa protein covalently linked to matrix metalloproteinase-9 (MMP-9) in human neutrophils [25]. It is highly induced proteins in the kidney after ischemic or nephrotoxic AKI in animal models [26]. Its levels can be detected in the plasma of patients as early as 2 h, peak at approximately 6 h after injury and its levels remain elevated for as long as five days, after which they begin to decrease [18].

The study has several limitations as it was conducted at a single center with a small sample size and a strict inclusion criterion of patients with SIRS. PNGAL was measured within 12 h of ICU admission; however measurement within 2–4 h may capture earlier changes that occur with kidney injury. However, it has been observed that there were no differences in NGAL performance when measured earlier within the ED or later in the ICU. A previous study, show plasma NGAL level, especially within 12 h of ICU admission, is an early and accurate predictor of AKI.

## Conclusion

High plasma NGAL levels noted within 12 h of ICU admission allows AKI diagnosis 48 h prior to the diagnosis based on RIFLE criteria and proves to be an early and accurate predictor of AKI. It can be utilized as an early biomarker for the detection of AKI in critically ill patients with sepsis in ICU so that potentially beneficial therapies can be initiated before irreversible kidney injury occurs.

## Abbreviations

AKI: Acute Kidney Injury
ARF: Acute Renal Failure
ATN: Acute tubular necrosis
AUC: Area under Curve
BMI: Body mass index
EDTA: Ethylene diamine-tetra-acetic-acid
GFR: Glomerular Filtrate rate
ICU: Intensive Care Unit
PNGAL: Plasma Neutrophil Gelatinase associated Lipocalin
RIFLE: Risk.Injury.Failure. End. Stage
SCr: Serum Creatinine
SPSS: Statistical package for social sciences
